# Central Nervous System Prophylaxis and Treatment in Acute Leukemias

**DOI:** 10.1007/s11864-022-01032-5

**Published:** 2022-12-13

**Authors:** Susan Y. Wu, Nicholas J. Short, Lewis Nasr, Bouthaina S. Dabaja, Penny Q. Fang

**Affiliations:** The University of Texas MD Anderson Cancer Center, 1515 Holcombe Blvd, Houston, TX 77030 USA

**Keywords:** CNS leukemia, Leukemia, Craniospinal radiation, Whole brain radiation, Intrathecal chemotherapy, High-dose methotrexate, Cytarabine, CAR-T, Stem cell transplant

## Abstract

Improvements in systemic therapy in the treatment of acute lymphoblastic leukemia (ALL) and acute myeloid leukemia (AML) have improved patient outcomes and reduced the incidence of CNS relapse. However, management of patients with CNS disease remains challenging, and relapses in the CNS can be difficult to salvage. In addition to treatment with CNS-penetrant systemic therapy (high-dose methotrexate and cytarabine), intrathecal prophylaxis is indicated in all patients with ALL, however is not uniformly administered in patients with AML without high-risk features. There is a limited role for radiation treatment in CNS prophylaxis; however, radiation should be considered for consolidative treatment in patients with CNS disease, or as an option for palliation of symptoms. Re-examining the role of established treatment paradigms and investigating the role of radiation as bridging therapy in the era of cellular therapy, particularly in chemotherapy refractory patients, is warranted.

## Introduction

Central nervous system (CNS) involvement in adult patients with acute leukemia is relatively uncommon and portends a poor prognosis. In this article, we review risk factors for CNS involvement, diagnostic criteria, prophylaxis, and treatment, with an emphasis on the role of radiation therapy (RT) as well as novel treatments including chimeric antigen receptor (CAR) T cell therapy.

## Overview and risk factors

In adult patients with acute leukemia, the CNS may be involved at the time of initial diagnosis or relapse and is associated with worse prognosis. With ongoing advances in systemic therapy, preventing CNS relapse, which may precede or occur concurrently with marrow relapse, has become increasingly important. This scenario requires dedicated CNS prophylaxis and therapeutic strategies, which remain a critical and unmet need. This section will discuss risk factors for CNS involvement in adult patients with ALL and AML.

In adult patients with ALL, 4–7% will have CNS involvement at diagnosis [1–3] compared to approximately 1–3% in AML [4, 5]. Across analyses of 11 clinical trials from the Eastern Cooperative Oncology Group-American College of Radiology Imaging Network (ECOG-ACRIN), CNS positivity at diagnosis of AML was not higher when lumbar puncture (LP) was mandatory compared to at the discretion of investigators (0.86% vs. 1.41%, *p* = 0.18), and did not appear to impact rate of initial complete response (CR) or overall survival (OS) [4], though some retrospective data suggest higher rates of cerebrospinal fluid (CSF) positivity with mandatory LP at diagnosis [6]. As such, the majority of data on prophylaxis and treatment is in the setting of ALL.

In the absence of CNS prophylaxis, over 30% of patients with ALL who achieve a complete response may develop CNS relapse, and risk has decreased in the modern era with better systemic control of disease [7, 8]. With modern regimens with improved CNS penetrance, including high-dose methotrexate and cytarabine and tailored intrathecal (IT) therapy, the risk of isolated CNS relapse is low and thus the toxicity and benefit of CNS prophylaxis must be weighed [9]. Among 439 patients with ALL treated at MD Anderson Cancer Center who achieved a complete response to vincristine, doxorubicin, dexamethasone (VAD) ± hyperfractionated cyclophosphamide, and tailored CNS prophylaxis with systemic or IT chemotherapy (without RT for CNS prophylaxis), 32 (7%) had CNS recurrence [10]. Patients with CNS relapse may experience concurrent or subsequent relapse in the bone marrow, and even with reinduction systemic therapy and CNS-directed therapy the prognosis for these patients is poor; in this older series prior to the development of more novel ALL-directed therapies, median survival was 6 months [10]. Although IT-chemotherapy (with addition of radiation in patients with neurologic deficits) was able to achieve a CNS CR in 30/32 patients (94%), ten (31%) experienced a second CNS recurrence.

Therefore, particularly in the setting of ALL, identifying the patients at greatest risk for CNS involvement who may benefit from prophylaxis is of critical importance. Increased risk of CNS involvement has been associated with elevated white blood cell count, elevated serum LDH, and elevated cell proliferation index at diagnosis. In adult patients with ALL, younger age, high white blood cell count, extramedullary disease, mature B cell or T cell immunophenotypes, Philadelphia chromosome positivity (*t*(9;22) leading to the BCR-ABL fusion gene) or BCR-ABL-like disease, and rearrangements in *KMT2A* may be associated with increased risk of CNS involvement [11, 12]. Traumatic lumbar puncture may be associated with increased risk of CNS relapse, a risk that may be countered with additional intrathecal chemotherapy [13, 14].

In patients with AML, the risk of CNS relapse is low, particularly with contemporary treatment regimens. Using induction and consolidation with regimens containing cytarabine and an anthracycline, followed by allogeneic stem cell transplant when feasible, CNS relapse occurred in 0.3% of patients, compared to marrow relapse in 51% [15]. In one older study, young age, increased white blood cell count, a prominent monocytic component, core-binding factor (CBF) AML (i.e., inversion 16 or t [8, 21]), chromosome 11q23 abnormalities, trisomy 8, and FLT3-ITD mutations may be associated with increased risk of CNS involvement. However, the use of high-dose cytarabine-based regimens for patients with CBF AML may mitigate the risk in this population. In a subsequent retrospective analysis of patients with non-CBF AML, age < 64 years, elevated LDH at presentation, and the presence of a *FLT3*-ITD mutation were independently associated with increased risk of CNS relapse [5, 16–18].

## Clinical presentation and diagnostic criteria

**Fig. 1:**
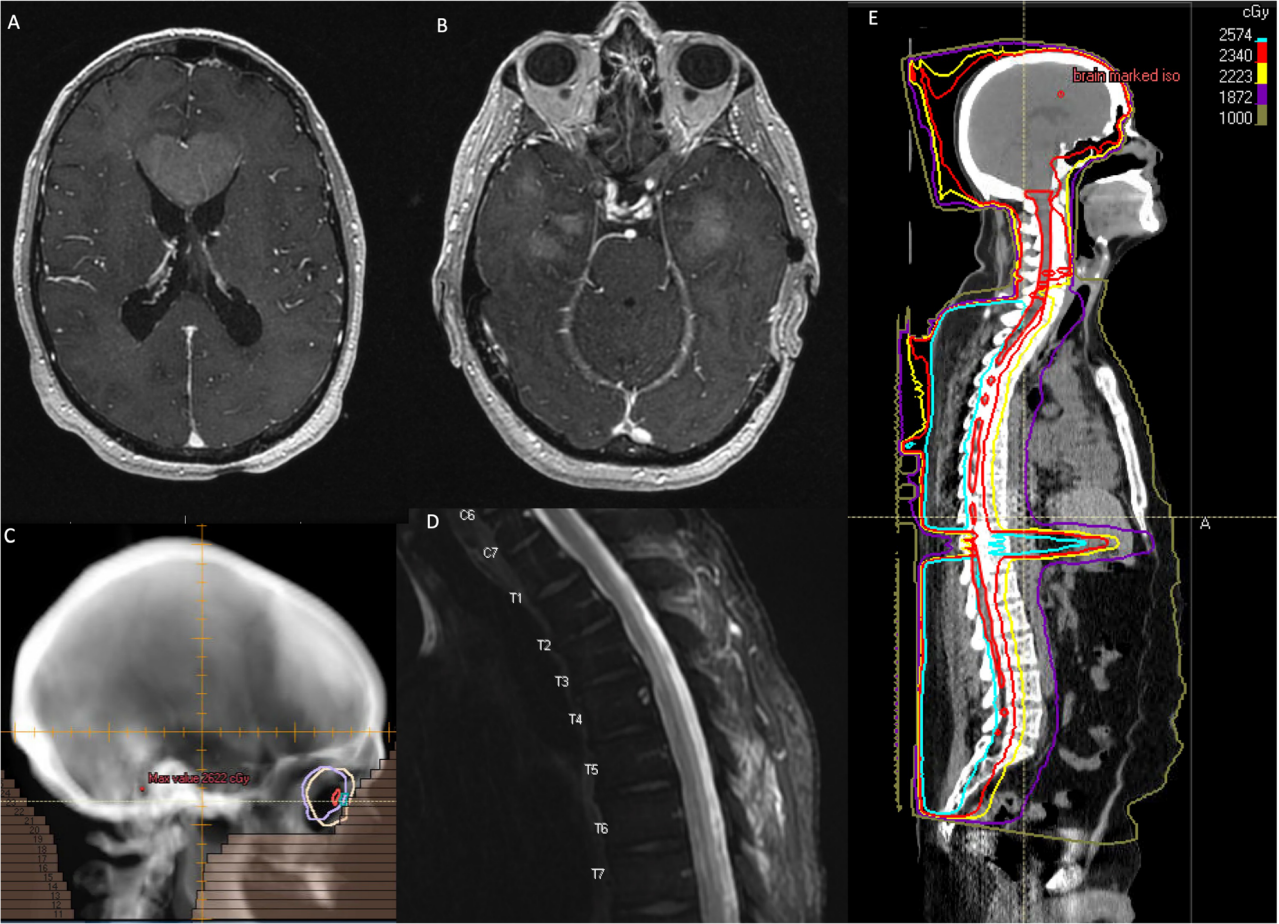
T1 post contrast axial images demonstrating **A**, a mass expanding the genu of the corpus callosum, measuring 4.4 × 2.7 cm, as well as **B**, multifocal lesions involving the anterior temporal lobes and leptomeningeal disease involving the cerebellar folia in a patient with CNS involvement from acute myeloid leukemia, **C**, treatment field for whole brain radiotherapy, **D**, T2 hyperintensity in the dorsal column of the thoracic cord concerning for treatment related toxicity in a patient with myelopathy and no evidence of CNS leukemia on lumbar puncture, and **E**, dose distribution for photon craniospinal radiation.

Clinical presentation of leukemic CNS involvement is highly variable. Patients may be asymptomatic, or have signs of increased intracranial pressure, focal cranial neuropathies, altered mental status, or evidence of cord compromise depending on the location of involvement. Workup in patients with symptoms of leukemic CNS involvement should include full neurologic exam, MRI of the brain (Fig. 1A, B) and spine, lumbar puncture (following imaging if concern for increased intracranial pressure), and in patients with ocular symptoms a thorough ophthalmologic assessment. Particularly in patients with limited leptomeningeal involvement, CSF cytology may be negative and the diagnosis may rely more heavily on imaging or flow cytometry/molecular studies [19].

Traditionally, CNS involvement is classified into three risk groups based on blasts and WBC in the CSF. CNS1 is defined as no blasts or WBC in the cytospin, CNS2 when blasts are present but fewer then 5 WBC per microliter are present, and CNS3 is defined as blasts in the setting of 5 or more WBC per microliter, or clinical signs of CNS involvement. In the setting of a traumatic lumbar puncture, with 10 or more red blood cells per microliter, the Steinherz/Bleyer algorithm is used to distinguish between CNS2 and CNS3 disease [20]. Due to limited cellularity in CSF samples, cytology has a high specificity (> 95%); however, more modest sensitivity (typically < 50%), which may result in false negatives [21]. Serial CSF sampling or high volume LP may improve diagnostic sensitivity [22].

As diagnostic techniques continue to improve, flow cytometry is a more sensitive modality for detecting CNS involvement in leukemia patients compared to traditional cytology [23–25]. In a large multicenter retrospective study of 240 patients with ALL, 43 patients had CNS disease identified by flow cytometry but not by conventional cytology [24]. The presence of CNS disease by flow cytometry may be associated with inferior disease free- and overall survival, and therefore it is recommended to perform flow cytometry in conjunction with conventional cytology whenever possible, particularly when the suspicion of CNS involvement is high [24].

## Prophylaxis

Optimal CNS prophylaxis in the setting of modern induction regimens with improved CNS penetrance is unclear, though use of high-dose (HD) systemic and intrathecal (IT) chemotherapy results in CNS relapse rates similar to regimens that include cranial radiation. Given concerns for long-term toxicity related to radiation, chemotherapy-based prophylaxis is often preferred.

Omura et al. randomized ALL patients who were in complete response (CR) following induction with prednisone, vincristine, and methotrexate, consolidation with cytarabine, thioguanine, asparaginase then vincristine/prednisone to CNS prophylaxis (with intrathecal methotrexate and cranial radiation (24 Gy in 12 fractions)) or no CNS prophylaxis [7]. Patients continued on 6-mercaptopurine, cyclophosphamide, and methotrexate maintenance. CNS prophylaxis was associated with a decreased risk of CNS relapse (11% vs. 32%, *p* = 0.03); however, hematologic remission and overall survival did not differ between the groups. The role of CNS prophylaxis was also explored in an analysis of 4 consecutive trials conducted at MD Anderson Cancer Center for patients with ALL, with (I) pre-VAD (no CNS prophylaxis), (II) VAD with high-dose systemic therapy for prophylaxis, (III) modified VAD with high dose systemic therapy for all patients and additional IT chemotherapy for high risk patients after achieving a CR, and (IV) hyperCVAD with early high dose systemic therapy and IT chemotherapy during induction (with 16 IT chemotherapy treatments for the high risk group vs. 4 for the low-risk group) [2]. High risk in this analysis was defined as elevated LDH or highly proliferative disease [26]. Across the four trials, 4% of patients had CNS involvement at diagnosis. The 3-year CNS event-free rate was significantly higher with hyper-CVAD, early high-dose systemic therapy, and risk-stratified IT-chemotherapy [2]. Of note, the benefit to CNS prophylaxis was only apparent in high-risk patients and 3-year CNS-leukemia-free survival was 33% pre-VAD, 70% for both VAD and modified VAD, and 98% for hyperCVAD. A benefit for CNS prophylaxis was not significant among low-risk patients, likely due to low incidence in this population even with inadequate prophylaxis. This analysis also suggests that early incorporation of IT chemotherapy during induction therapy may be beneficial.

Different intrathecal prophylaxis regimens have been explored in ALL, including triple intrathecal therapy (methotrexate, cytarabine, prednisone) which has been demonstrated to decrease CNS relapse however without improvement in EFS compared to IT methotrexate alone in pediatric ALL patients [27]. At our institution, single agent IT-chemotherapy is used in the setting of prophylaxis with methotrexate (12 mg if by LP, 6 mg by Ommaya) or cytarabine (100 mg), while triple intrathecal chemotherapy (hydrocortisone 50 mg, cytarabine 40 mg, and methotrexate 12 mg) is typically used to treat CNS leukemia and is delivered twice weekly until the CSF is negative, weekly for 4 weeks, then every other week for 4 weeks, and then monthly for approximately 4 months. The number of IT chemotherapy treatments at MD Anderson has evolved over time for low, intermediate, and high risk patients [28]. Currently, the majority of patients at our institution with Philadelphia chromosome-negative B cell ALL and T cell ALL receive 8 doses of IT chemotherapy [29•]. In patients with newly diagnosed Philadelphia chromosome-positive ALL however, 12 IT chemotherapy treatments has been shown to decrease the risk of CNS relapse compared to 8 and is our current practice [30]. In Burkitt leukemia, prophylaxis is often intensified to 16 IT chemotherapy treatments [31]. To avoid simultaneous administration of IT and high-dose systemic methotrexate during even cycles of hyperCVAD, the sequence of IT chemotherapy is reversed during even cycles (cytarabine on day 2 ± 2, and methotrexate on day 8) [29•]. Notably, with the development of novel regimens with reduced doses of chemotherapy or even chemotherapy-free regimens (e.g., blinatumomab plus a tyrosine kinase inhibitor for Philadelphia chromosome-positive ALL), it is uncertain whether more intensive IT prophylaxis might be needed.

Routine prophylaxis in AML is generally not indicated due to the overall low risk of CNS relapse, although it may be considered in certain clinical scenarios [32]. Modern induction and consolidation regimens with stem cell transplant for AML are associated with CNS relapse rates < 1% [15]. In this large retrospective series, LP was not routinely performed at diagnosis in patients without clinical suspicion for CNS involvement, and IT prophylaxis was only used in patients with acute myelomonocytic or acute monocytic leukemia with leukocytosis. IT prophylaxis was not associated with a difference in CNS relapse. Similar results demonstrating no significant benefit to IT prophylaxis have been reported in additional series of AML patients even in the setting of hyperleukocytosis ≥ 100,000 cells/microL [33]. At our institution, we generally administer 2 doses of prophylactic IT cytarabine for patients with AML and one or more high-risk features: WBC ≥ 50 × 10^9^/L, elevated LDH, and/or a *FLT3*-ITD mutation. Patients with *KMT2A*-rearranged AML may have a particularly high risk of CNS relapse, and therefore we typically give doses of IT cytarabine to these patients, although robust data to support this practice are lacking.

## Role of radiation

The primary role of radiotherapy in the management of CNS leukemia is at the time of CNS relapse. These data are primarily in the setting of pediatric acute leukemia and have been extrapolated to adults. Meta-analysis of 10 cooperative study group trials of pediatric patients with ALL demonstrated that pre-emptive cranial radiation was only associated with improved rate of CNS relapse in patients with CNS3 disease, with no difference in overall survival [34]. There was no difference in the rate of 5 year CNS relapse, any event, or death with pre-emptive cranial radiation in any other group. Contemporary trials have omitted cranial radiation in the treatment of pediatric ALL even in patients with high risk disease; by increasing the intensity of systemic and intrathecal chemotherapy, the 5-year continuous complete remission rate on the Total Therapy Study XV was higher among patients who would have met criteria for CNS radiation but were treated without, compared to historic controls [35]. In adult patients with ALL, Cancer and Leukemia Group B (CALGB) 19802 was a phase 2 study that evaluated the efficacy of intensified daunorubicin and cytarabine, and also assessed if high dose systemic and intrathecal methotrexate could replace cranial radiotherapy (CRT) for CNS prophylaxis [36]. This study demonstrated a 6% risk of isolated CNS relapse, lower then prior CALGB studies that included radiation. This was also consistent with the results published by the group at MD Anderson, which demonstrated that with hyper-CVAD alternating with high-dose methotrexate and cytarabine, IT-chemotherapy with risk-adapted maintenance therapy and no prophylactic CRT, the rate of CNS relapse in patients without initial CNS leukemia was low at 4% [9]. In this series, 17/19 patients with initial CNS involvement achieved systemic and CNS remission, though patients with cranial nerve involvement were permitted to receive radiation to a dose of 24–30 Gy in 10–12 fractions to the skull base or whole brain.

Given the increased risk of CNS relapse in patients with a history of CNS leukemia, and in particular CNS3 disease [37], studies suggest a role for radiation in this scenario. In a retrospective series of adults undergoing hematopoietic cell transplant for AML at Fred Hutchinson Cancer Research Center, 71 had positive CSF cytology prior to transplant [38]. Of these 71 patients, 52 received IT-chemo alone while 19 received IT chemotherapy and radiation (cranial or craniospinal at the discretion of the treating physician; IT chemotherapy regimens and number of treatments were not different in CNS positive patients who did or did not receive RT). While patients with CNS involvement who received IT chemotherapy alone had inferior 5-year relapse-free-survival of 6% compared to patients without CNS involvement (35%), those who received IT chemotherapy and RT demonstrated RFS of 32% even after controlling for variables such as disease status and 12 Gy TBI as part of conditioning. Similar results were seen with regard to 5-year overall survival, which was significantly lower in patients with CNS disease who received IT chemotherapy alone (6%) compared to those who also received RT (42%).

In the era of CNS-penetrant therapy for ALL, CNS prophylaxis following allogeneic stem cell transplant is controversial and practice patterns vary, with some institutions offering prophylaxis for patients only with a prior history of CNS disease, and others for all patients [39]. This multi-institutional study found that CNS relapse following transplant was uncommon (4%), and while more likely in those with a history of prior CNS leukemia, there was no benefit for post-transplantation CNS prophylaxis with IT chemotherapy and/or radiation. The intensity of transplant conditioning also did not impact CNS relapse rate following transplant. Intrathecal chemotherapy following stem cell transplant may also be associated with a higher incidence of leukoencephalopathy [40]; however, patients receiving post-transplant IT prophylaxis were typically higher risk patients or those with prior CNS involvement and may have received more cumulative CNS-directed therapy prior to transplant.

Radiation can be considered in patients who need urgent palliation, although it must be noted that responses to systemic or intrathecal chemotherapy, or steroids, can be rapid as well. In the setting of palliation, focal RT can often be beneficial, particularly for patients who have been refractory to chemotherapy [41]. In a series of 163 patients, 2/3 of whom presented with cranial neuropathy, radiation was associated with resolution or improvement of deficits in almost 70% of patients. With regards to treatment field, 12-month CNS progression-free survival was lower in patients who received focal skull base RT compared to more comprehensive fields (whole brain or craniospinal) (51% vs. 77%, *p* = 0.02) [42]. In this series, 77% of patients had positive pathology (CSF or biopsy), and 57% had imaging findings consistent with CNS involvement on CT or MRI. The most common RT dose was 24 Gy, and dose was not significantly different across different treatment fields (*p* = 0.55). In patients with CNS relapse after CR to induction therapy, radiation has been associated with a lower risk of second CNS relapse (2/15 or 13% compared to 8/17 or 47% in patients who did not received RT, *p* = 0.06) [10].

At our institution, we favor comprehensive CSI in patients with CNS relapse, particularly if planned for curative-intent hematopoietic stem cell transplant or CAR T-cell therapy (discussed below). Patients should complete CSI prior to conditioning for hematopoietic stem cell transplant or lymphodepleting chemotherapy prior to CAR-T. In this setting, the dose for CSI is typically 23.4 Gy in 13 fractions both for consolidation and to treat gross disease. Focal radiation or whole brain RT can be considered in patients with poor performance status or requiring urgent palliation, particularly in the setting of chemotherapy-refractory disease.

## Toxicity considerations

New neurologic deficits should prompt comprehensive imaging and pathologic assessment to differentiate between treatment-related toxicity and symptomatic CNS leukemia. Myelopathy has been documented in patients receiving systemic and intrathecal chemotherapy in the absence of RT [43–46], although it is more commonly associated with combined modality therapy. The toxicity, based on our published data, appears related to simultaneous delivery of intrathecal and systemic high-dose MTX, thereby overloading the mechanism for clearing MTX in the CSF. We recommend avoiding IT MTX within 48 h of high-dose MTX and have adjusted the intrathecal chemotherapy schedule of our hyper-CVAD regimen accordingly [29•].

Neuroimaging findings suggestive of treatment-related toxicity include diffuse periventricular white matter hyperintensity on T2-weighted MRI. Metabolic imaging or advanced MRI techniques, such as spectroscopy or perfusion-weighted imaging, may be helpful in distinguishing treatment related toxicity from disease progression. Autopsy evaluation of 5 patients with treatment-related leukoencephalopathy and no evidence of disease demonstrated myelin and axonal loss, gliosis, spongiosis and rarefaction of white matter, and tissue necrosis correlating with areas of enhancement on MRI [47]. In patients with spinal cord myelopathy related to treatment, MRI imaging typically demonstrates T2-hyperintensity, often involving the dorsal columns (Fig. 1D) [48]. Symptoms and imaging may be progressive, and MRI may initially be unremarkable. In the setting of cord toxicity, pathologic evaluation may demonstrate necrosis particularly of the grey matter with infiltration of macrophages and lymphocytes [46].

In a series of 13 leukemia patients who developed myelopathy following CNS-directed therapy at MD Anderson Cancer Center (median 17 intrathecal treatments, without therapeutic RT to the spine), 7 patients (54%) had MRI imaging initially read as normal though on re-review the majority of these patients had subtle findings [48]. In this series, patients presented with ascending lower extremity paresthesias, incontinence, and weakness a median of 15 days following last IT-methotrexate. Myelin basic protein was elevated in all assessed patients.

Prospective MRI evaluation of children receiving high-dose and intrathecal methotrexate demonstrated leukoencephalopathy in 23% of patients. Leukoencephalopathy on MRI after consolidation was 100% sensitive for neurotoxic events, though the positive predictive value was only 13% and 8/14 patients developed symptoms prior to MRI findings [49]. In 77% of patients who developed radiographic leukoencephalopathy, MRI abnormalities were still evident at week 120.

In general, RT should not be delivered concurrently with high-dose systemic or intrathecal chemotherapy. In a series of 23 patients with toxic myelopathy, 13 received radiation and CNS directed therapy with IT or HD methotrexate or cytarabine, with death in 6 patients of whom 5 had pathologic evidence of cord necrosis, with ventilator dependence in an additional 2 and paralysis in an additional 4 patients [50]. In this series, myelin basic protein was elevated in all assessed patients, and preceded neurologic symptoms in one patient in whom this was being followed prospectively. Due to the concern for toxicity, a washout of 2 weeks following HD methotrexate or cytarabine and RT is preferred; however, when urgent palliation is indicated, RT can be considered after 48–72 h [51•]. Ideally, CNS-directed chemotherapy should be completed prior to radiation, as radiation prior to chemotherapy may increase toxicity [52].

Patients receiving combined modality therapy with chemotherapy (IT ± IV) and radiation are more likely to experience cognitive decline following treatment, particularly if treated at an early age, even with moderate doses of radiation [53]. Historically, in the setting of adult patients with primary CNS lymphoma treated with methotrexate (2.5 g/m^2^), vincristine, procarbazine, and intrathecal methotrexate (12 mg), followed by whole brain radiotherapy (WBRT, initially to 45 Gy) and high dose cytarabine after RT, severe delayed neurological toxicity was seen in 15% of patients, which was fatal in 10% of patients [54]. The risk of fatal neurotoxicity was increased in patients 60 years of age or older (16% vs. 6% if younger). However, retrospective review of 185 patients treated in the era of lower WBRT doses (23.4 Gy in 1.8 Gy fractions) suggests the risk of treatment related neurotoxicity at 3 years may not be higher in patients receiving WBRT after HD-MTX-based therapy compared to those receiving HD-MTX based therapy alone (20.2% vs. 21.2%, *p* = 0.63) [55]. This data has established the standard dose of 23.4 Gy as effective with limited neurotoxicity even with long follow up.

In situations where there is preexisting neurotoxicity, the addition of RT can worsen clinical outcomes. In the series by Pinnix et al., two patients with myelopathy following CNS-directed chemotherapy received additional radiation due to concern for leukemic involvement [48]. Post-mortem exam of one patient demonstrated degeneration in the dorsal column in the unirradiated cord; however, prominent necrosis and parenchymal hemorrhage was evident in the irradiated portion of the cord.

Blinatumomab is a bispecific T cell receptor engaging antibody that binds CD3^+^ T cells and CD19^+^ lymphoblasts and is highly effective in relapsed/refractory B cell ALL and can also eradicate measurable residual disease in these patients [56, 57]. However, blinatumomab is associated with CNS toxicity in up to half of patients, mostly grade 1 or 2 [56]. Neurologic symptoms from blinatumomab may include paresthesias, aphasia, confusion, tremor, or ataxia, which typically improve with discontinuation of the medication. Generally, our practice is to avoid concurrent RT with blinatumomab. However, the half-life of blinatumomab is short (2.11 h), so RT may be administered sequentially if indicated.

At our institution, we favor completion of CNS-directed systemic or IT chemotherapy prior to radiation, with a washout of ideally 2 weeks following high-dose methotrexate. A shorter washout period can be considered following blinatumomab or IT chemotherapy, particularly with cytarabine which has a half-life of approximately 3.4 h [58, 59].

### Considerations in patients with neurologic deficits without clear evidence of leukemic involvement

In patients without clear evidence of leukemia on CSF cytology or imaging, treatment-related toxicity must be carefully considered, particularly prior to RT. CSF analysis should include myelin basic protein (MBP) level, which is often elevated in the setting of active demyelination [60]. MBP may be elevated both in the setting of acute MTX-related toxicity or delayed neurotoxicity from IV or IT chemotherapy ± RT [61]. Additional contributing causes of myelopathy should be evaluated, including serum folate, B12, homocysteine, and methylmalonic acid levels. Repletion of B12 and folate, as well as administration of dextromethorphan, can be considered though neurologic deficits may not improve [48]. In patients undergoing WBRT, memantine has been shown in a randomized controlled trial to improve cognitive function over time with comparable toxicity compared to placebo [62]. Based on this trial, our institutional practice is to begin memantine ideally on the first day but at least within 3 days of starting WBRT, initially at 5 mg/day and increasing by 5 mg/day each week to a goal dose of 20 mg/day, for a duration of 6 months.

## Radiation treatment techniques

Target volumes for craniospinal radiation (CSI), whole brain RT (WBRT), and treatment planning considerations have been described elsewhere and will only be discussed briefly here (Fig. 1C, E) [51•, 63]. WBRT fields typically include the whole brain, extended inferiorly to C1 or C2 and anteriorly to include at least the posterior two-thirds of the orbital globes. Plans should be reviewed to ensure coverage of the cribriform plate, skull base, and middle cranial fossa. Treatment is typically delivered with opposed lateral 6 MV beams, though depending on the clinical scenario physicians may prefer to rotate the gantry to match the anterior field edge to limit dose to the lenses.

Given the CSF space is contiguous, more comprehensive targets may be associated with improved CNS PFS [42] or DFS [64] and should be considered particularly in high-risk patients prior to transplant. In CSI planning, the brain, spinal cord, and sacral nerve roots (typically terminating at S2-S3) are targeted. In adults, this results in long treatment fields which poses unique challenges of matching multiple fields. Techniques for managing junctions vary by institution, however, may involve feathering and field-in-field planning to limit hot spots in the areas of overlap. Patients can be treated prone or supine, each with their own advantages. The light fields can be visualized directly on the patient if treating in the prone position, while x-rays can confirm patient position in the supine position. No significant dosimetric differences with regards to coverage, homogeneity, or dose to organs at risk have been demonstrated between the supine or prone position [65], though the supine position is more comfortable for patients and is favored for patients requiring anesthesia for airway access. Use of proton-based CSI may improve toxicity related to RT by limiting anterior dose to organs at risk [66–68]; however, also poses unique challenges due to increased relative biologic dose at the distal edge of the Bragg peak and beam range uncertainty. Although more common for children, myeloablative total body irradiation (TBI) may be used in younger adult patients prior to allogeneic transplant. If craniospinal radiation is indicated in these patients, the CSI dose should be adjusted such that the cumulative dose to the brain and spinal cord does not exceed approximately 24 Gy.

### CAR T cell therapy

CD19-specific CAR T cell therapy has demonstrated high complete response rates of 70–80% in patients with relapsed or refractory (r/r) B-ALL [69, 70]. Although patients with symptomatic CNS leukemia are often excluded from clinical trials, analysis of 52 patients with CNS3 disease at the time of relapse or within 30 days of screening for CD19 CAR T cell therapy (23% received CD22 CAR T cells as well) demonstrated CNS remission in 85% of patients [71]. Importantly, CNS remission was higher in patients who received CNS-directed bridging therapy (systemic or intrathecal chemotherapy (33% of whom went to CAR-T with CNS3 disease)) compared to those who did not (all of whom went to CAR-T with CNS3 disease). Grade 3–4 neurotoxicity was also associated with higher pre-infusion CNS disease burden. In a post-hoc analysis of 195 patients with relapsed or refractory ALL from five clinical trials in which participants received CD19-directed CAR T cell therapy, patients with isolated CNS involvement had significantly higher overall survival at 2 years compared to those with CNS disease and bone marrow involvement (95% CI 91% [82–100] vs. 71% [64–78]; *p* = 0.046) [72]. Consideration of CNS directed therapy such as radiation, particularly in patients who have chemo-refractory disease, may be even more compelling in order to achieve durable CNS control in patients with isolated CNS disease [72]. In a phase I/II trial of CD19 CAR T cell therapy for r/r B cell ALL, 17 patients had CNS involvement of whom 77% were able to achieve a CR, though this was lower than in patients without CNS involvement [73]. In this study, there was no difference in incidence or severity of neurotoxicity in patients with or without CNS leukemia. Additional protocols with small numbers of patients with CNS involvement and case reports have similarly demonstrated that CD19 CAR T cell therapy can be effective for patients with CNS leukemia [74, 75]. Investigation of radiation treatment as a bridging strategy to decrease CNS disease burden prior to CAR-T cell therapy is warranted.

## Conclusion

CNS leukemia portends poor prognosis and multidisciplinary evaluation is essential. Prevention of CNS relapse is the most important component of therapy and should consist of risk-adapted prophylactic IT chemotherapy for all patients with ALL and in some patients with AML with high-risk disease features. Durable control of disease, particularly in patients with isolated CNS relapse, is increasingly important in the era of targeted and cellular therapies. For patients who relapse with CNS leukemic involvement, radiation treatment can be an effective treatment for CNS consolidation after chemotherapy and for palliation of symptomatic disease. Investigation of RT as potential bridging therapy to CAR-T is warranted, particularly for patients with chemo-refractory disease.
